# Temporal trends in lung cancer and radon gas exposure: an ecological study in Rio Grande do Sul

**DOI:** 10.3332/ecancer.2025.2033

**Published:** 2025-11-14

**Authors:** Jacson C Jung, Juvenal S D Costa, Christie H E Oliveira, Oderson A S Filho, Vera M V Paniz

**Affiliations:** 1 Postgraduate Program in Collective Health, University of Vale do Rio dos Sinos – UNISINOS, São Leopoldo, RS 93022-750, Brazil; 2 Postgraduate Program in Geology, University of Vale do Rio dos Sinos – UNISINOS, São Leopoldo, RS 93022-750, Brazil; 3 Brazilian Geological Survey - SGB, Center of Applied Geosciences, Rio de Janeiro Office, Rio de Janeiro, RJ 22290-255, Brazil

**Keywords:** ecological studies, incidence, lung neoplasms, radon, time series studies

## Abstract

**Objective:**

This study aimed to compare lung cancer incidence, hospitalisation and mortality rates among municipalities of Rio Grande do Sul from 2000 to 2021 based on estimated radon gas exposure.

**Methods:**

An ecological time series study was conducted using data from the Brazilian Geological Survey and the Ministry of Health. Geoprocessing techniques and Prais-Winsten regression were employed for data analysis.

**Results:**

No statistically significant differences were found in average outcome rates across the study period when grouped by radon exposure probability. Municipalities with higher exposure probabilities showed an increasing trend in lung cancer incidence and hospitalisation rates, while mortality remained stable. Additionally, hospitalisation rates increased in municipalities with lower exposure probabilities.

**Conclusion:**

This study demonstrated the methodological challenges of investigating the association between gases and cancer etiology.

## Introduction

Lung cancer is the second most common cancer globally, with an estimated 2.3 million new cases diagnosed in 2020, accounting for 11.4% of all cancer cases. This cancer type remains the leading cause of cancer-related mortality, with an estimated 1.8 million deaths (18%) in the same year [1].

In Brazil, time-series studies [2–4] have consistently identified lung cancer as the most prevalent neoplasm, with a higher incidence in women [2]. It also represents the primary cause of cancer-related deaths, surpassing breast and prostate cancers. In 2020, lung cancer accounted for 35,160 cases in Brazil, representing 13.5% of all cancers in the country [5]. The southern region of Brazil experienced a significant increase in lung cancer mortality rates between 1996 and 2015, with Rio Grande do Sul reporting the highest rates [6].

Although smoking rates have declined in Brazil, has decreased, tobacco-smoking remains the primary risk factor for lung cancer, accounting for 80%–90% of diagnosed cases. Exposure to mineral dust, asbestos, silica and pesticides also increases the risk of lung cancer [7–9]. Additionally, radon gas is considered a major cause of lung cancer among non-smokers [10–16].

Radon is a colorless, odorless and tasteless radioactive gas that forms naturally from the radioactive decay of uranium and thorium, which are found in trace amounts in rocks, soil and water. Uranium decays into radium, which subsequently transforms into radon [13, 17]. While direct data on radon gas emissions in Rio Grande do Sul are limited, estimates can be made based on uranium equivalent (eU) data from geophysical surveys [17, 18].

Consequently, this study aimed to compare lung cancer incidence, hospitalisation and mortality rates among municipalities in Rio Grande do Sul from 2000 to 2021, considering the probability of radon gas exposure.

## Methods

This ecological time-series study [19] analysed lung cancer incidence, hospitalisation and mortality rates among municipalities in Rio Grande do Sul, Brazil, from 2000 to 2021, based on estimated radon exposure. Rio Grande do Sul is a southern Brazilian state with 497 municipalities, including its capital, Porto Alegre. Its borders include Santa Catarina, Argentina, Uruguay and the Atlantic Ocean, covering 281,707.151 km^2^ (the ninth largest state by area in Brazil) and having a population of 10,882,965 in 2022 (the sixth largest state by population in Brazil). Its demographic density is 38.63 inhabitants/km^2^ (25th densest state in the country) and its human development index in 2021 was 0.771 (fifth most developed state in Brazil) [20].

The Rio Grande do Sul Shield Aerogeophysical Project [21], conducted by the Geological Survey of Brazil, mapped geophysical aspects within the region defined by UTM 21S (central meridian 57º W GR) and UTM 22S (central meridian 51º W GR). This survey analysed 85 municipalities in the state. Only municipalities with 100% of their territory mapped through aerogammaspectrometry were included in this study, resulting in a reduced sample of 37 municipalities. To identify municipalities with the highest radon gas exposure, eU data were used as predictors. eU data were obtained from the Geological Survey of Brazil through the Rio Grande do Sul Shield Aerogeophysical Project (available at: https://geosgb.sgb.gov.br/). Spatial analysis was performed using QGIS Desktop version 3.22.5, with the SIRGAS 2000 geodetic coordinate system. eU values per municipality were obtained through geoprocessing by intersecting aerogammaspectrometry geophysical data with municipal territorial boundaries.

Initially, municipalities were classified based on their maximum detected eU values into categories of normal and high radon exposure probability. Uranium concentrations in the Earth’s crust typically range from 2.0 to 4.0 parts per million (ppm) [18, 22]. Thus, municipalities with maximum eU values of up to 4.0 ppm were considered to have normal concentrations, while those exceeding this value were classified as having elevated uranium concentrations, corresponding to a higher probability of radon gas exposure. Subsequently, to further refine this classification, municipalities with maximum eU values greater than 4.0 ppm were stratified according to the median of the eU values observed within their territory. The choice of the median was motivated by the spatial heterogeneity of the data, allowing for a more robust representation of uranium concentration in each municipality. As a result, the following municipalities were categorised as having a low probability of radon exposure: Amaral Ferrador, Arroio do Padre, Caçapava do Sul, Cachoeira do Sul, Candelária, Candiota, Canguçu, Cerrito, Cerro Branco, Cerro Grande do Sul, Dilermando de Aguiar, Formigueiro, Hulha Negra, Lavras do Sul, Morro Redondo, Novo Cabrais, Pantano Grande, Paraíso do Sul, Pedro Osório, Pinheiro Machado, Piratini, Restinga Sêca, Santa Margarida do Sul, Santa Maria, Santana da Boa Vista, São João do Polêsine, São Sepé, Sentinela do Sul, Sertão Santana, Silveira Martins, Vera Cruz and Vila Nova do Sul. In contrast, Arroio dos Ratos, Barão do Triunfo, Chuvisca, Dom Feliciano and Encruzilhada do Sul were considered to have a high probability of radon exposure.

This study examined lung cancer incidence, hospitalisation and mortality rates. Lung cancer was defined using code C34 from the 10th Revision of the International Statistical Classification of Diseases and Related Health Problems, which refers to malignant neoplasms of the bronchi and lungs.

Lung cancer data were obtained from the Unified Health System’s Information Technology Department (DATASUS) using TabNet, organised by ‘municipality of residence.’ New lung cancer cases were extracted from the ‘Epidemiology and Morbidity’ field, specifically the ‘Time until the start of oncological treatment-PANEL-oncology’ item (available from 2013). Hospitalisations for lung cancer between 2000 and 2021 were sourced from the Unified System of Health (SUS) Hospital Morbidity System (SUS/SIH). Death information was obtained from the Mortality Information System (SIM), specifically the ‘Vital statistics’ field, ‘Mortality-since 1996 by ICD-10’ sub-item ‘General mortality’ (2000–2021). Population data was also sourced from DATASUS. Cases, hospitalisations and total deaths were collected by municipality, year and age range.

Age-standardised coefficients were calculated using the direct method [19] to control for differences in age profiles across populations. The total population of Rio Grande do Sul in 2010 was used as the standard, and coefficients were expressed as annual rates per 100,000 inhabitants.

Average coefficients and their corresponding 95% confidence intervals were calculated for each lung cancer outcome (incidence, hospitalisation and mortality) over each time period, stratified by radon exposure probability. To assess differences in average rates between municipalities based on radon exposure, Student *t*-tests were conducted with a statistical significance level of *p* < 0.05.

Temporal trend analysis was conducted using Prais–Winsten regression to address first-order autocorrelation. The analysis assesses whether outcomes exhibited stability, growth or decline over the study period. Results with *p*-values greater than 0.050 were considered stationary, while those with *p*-values ≤0.050 indicated increasing (positive coefficient) or decreasing (negative coefficient) trends. To enhance stability, moving averages were calculated every 5 years by combining average coefficients. Data processing and statistical analyses were performed using Microsoft Excel 2019^®^ and Stata 12 (StataCorp Lp@, College Station, TX, USA).

This study is part of a larger project titled ‘Residential Radon and Lung Cancer in the State of Rio Grande do Sul: Exposure Risk Map.’ In compliance with Resolution No. 466/2012 of the National Health Council, the main project was submitted to the Research Ethics Council of the University of Vale do Rio dos Sinos and approved with opinion No. 4,914,148 on 18 August 2021. This study utilised publicly available, de-identified data, as recommended by Resolution No. 510 of the National Health Council, passed on 7 April 2021. Therefore, informed consent was not required.

## Results

The lung cancer coefficients of 37 municipalities in Rio Grande do Sul were analysed. Of these, 32 (86.5%) were considered to have low radon exposure probabilities, while five (13.5%) were considered to have high exposure probabilities.

Between 2013 and 2021, no statistically significant differences were observed in average incidence rates (*p* = 0.720) between municipalities with low and high radon exposure probabilities (Table 1). Similarly, there were no significant differences in average hospitalisation (*p* = 0.821) or total mortality rates (*p* = 0.203) from 2000 to 2021 (Table 1).

**Figure 1.**
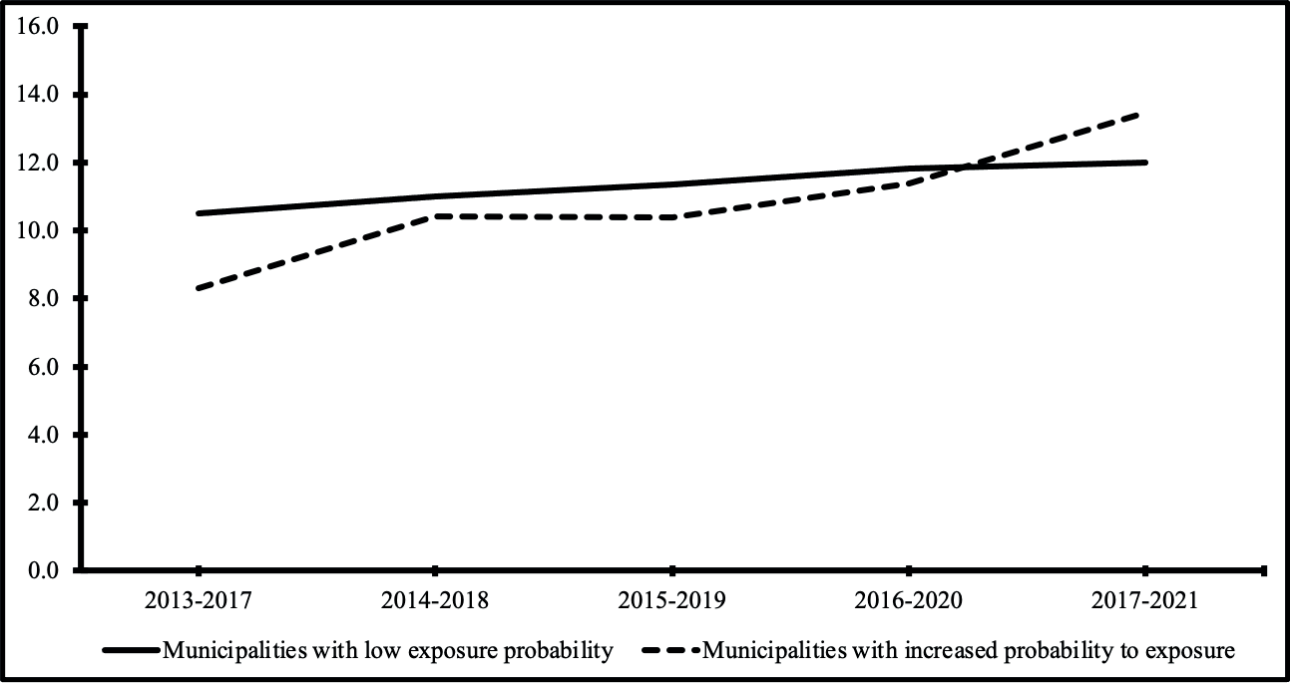
Standardised lung cancer incidence rates in the State of Rio Grande do Sul, Brazil (2013–2021), by estimated probability of radon exposure. This figure presents 5-year moving average lung cancer incidence rates standardised by age for 37 municipalities in Rio Grande do Sul. Municipalities were grouped according to their estimated probability of radon exposure, as inferred from airborne gamma spectrometry data (equivalent uranium – eU levels): – Low exposure probability: municipalities with lower eU levels. – Increased exposure probability: municipalities with higher eU levels, indicating greater radon susceptibility.

Temporal trend analysis revealed a stable lung cancer incidence trend (*p* = 0.153) in municipalities with low radon exposure probabilities and an increasing trend (*p* = 0.020) in municipalities with high probabilities (Table 2). This was confirmed by moving average calculations, which showed an upward trend in projected coefficients for municipalities with higher radon exposure probabilities from 2013 to 2021 (Figure 1).

**Figure 2.**
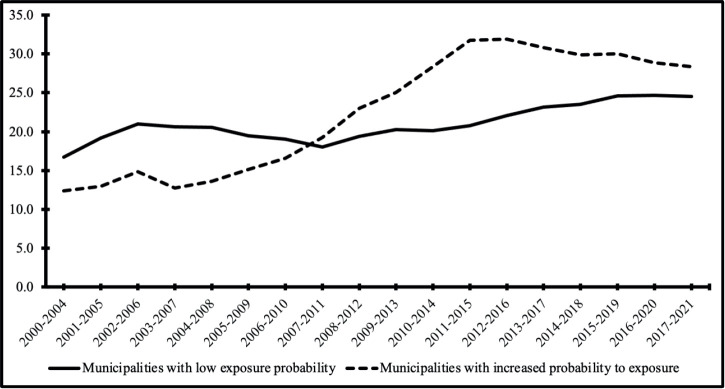
Standardised rates of hospitalisations for lung cancer in the State of Rio Grande do Sul, Brazil (2000–2021), by estimated probability of radon exposure. This figure shows 5-year moving average hospitalisation rates for lung cancer, standardised by age, in 37 municipalities of Rio Grande do Sul. Municipalities were classified based on the estimated probability of radon exposure derived from airborne gamma spectrometry (equivalent uranium – eU levels):– Low exposure probability: municipalities with lower eU levels. – Increased exposure probability: municipalities with higher eU levels, indicating greater radon susceptibility.

For hospitalisation rates, an increasing trend was observed in both municipalities with low (*p* = 0.021) and high (*p* <0.01) exposure probabilities (Table 2). Moving averages analysis also indicated an increasing trend in hospitalisation rates for municipalities with higher radon exposure probabilities, starting around 2006 (Figure 2).

**Figure 3.**
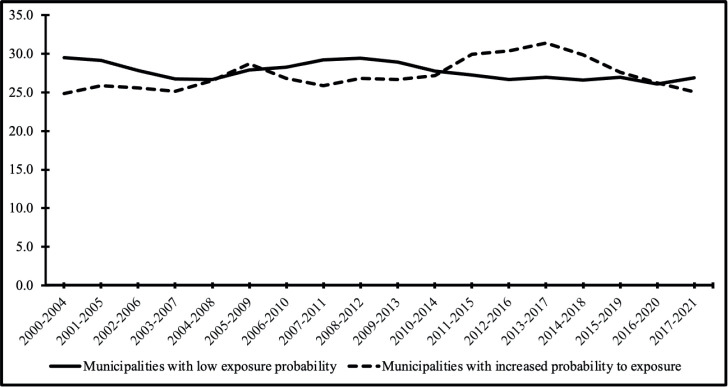
Standardised total mortality rates from lung cancer in the State of Rio Grande do Sul, Brazil (2000–2021), by estimated probability of radon exposure. This figure presents 5-year moving average age-standardised mortality rates from lung cancer in 37 municipalities of Rio Grande do Sul. Municipalities were categorised according to the estimated probability of radon exposure, based on airborne gamma spectrometry (equivalent uranium – eU levels): – Low exposure probability: municipalities with lower eU levels. – Increased exposure probability: municipalities with higher eU levels, indicating greater radon susceptibility.

Regarding total mortality rates, a stationary trend was observed in both municipalities with low (*p* = 0.058) and high (*p* = 0.899) radon exposure probabilities (Table 2). Moving average trends for this variable were similar for both groups of municipalities (Figure 3).

## Discussion

This ecological time-series study analysed lung cancer incidence, hospitalisation and total mortality rates in Rio Grande do Sul, Brazil, from 2000 to 2021, based on estimated radon exposure. Although no significant differences were found in average rates between municipalities with low and high radon exposure probabilities, temporal trends revealed important disparities. Municipalities with low radon exposure probabilities exhibited stable rates of lung cancer incidence and total mortality. Conversely, areas with higher radon exposure probabilities showed notable increases in lung cancer incidence and hospitalisation rates, while total mortality remained stable.

While this study did not establish a direct causal association between radon exposure and lung cancer incidence, previous systematic reviews and meta-analyses have done so [13–16]. The ecological study design used in this research limits the ability to definitively determine causality. However, the observed increasing trends in lung cancer incidence and hospitalisation rates among municipalities with higher radon exposure probabilities align with prior literature. While an environmental definition of radon gas exposure related to cancer occurrence has not been established, a linear dose-response relationship has been suggested [23]. Therefore, a potential association between higher radon exposure probabilities and increased lung cancer rates cannot be ruled out.

It is also important to consider other behavioural and environmental factors that may have influenced lung cancer trends during the study period. Tobacco smoking remains the primary risk factor, accounting for over 80% of cases [7, 8, 12]. Although its prevalence has declined in Brazil in recent decades, regional variations persist and the disease's long latency period may reflect past exposures [8]. In addition to smoking, air pollution in urban and industrial environments, particularly fine particulate matter [7, 12], is recognised as carcinogenic and may interact with radon exposure, increasing lung cancer risk. While these factors could not be measured within the adopted ecological design, they should be addressed in future investigations using individual-level data. Further investigation through targeted studies that consider other relevant risk factors in these municipalities is warranted.

Another relevant point is the impact of advances in diagnostic methods and lung cancer screening over time. It is important to highlight that in Brazil, there is currently no guidance for systematic lung cancer screening. The recommendation of the National Cancer Institute [7], corroborated by guidelines from the Brazilian Society of Thoracic Surgery, the Society of Pulmonology and the School of Radiology, consists of low-dose computed tomography screening for high-risk individuals, including current or recent ex-smokers (within the last 15 years), aged between 50 and 80 years, with a smoking history of at least 20 pack-years [7, 8]. The adoption of this recommendation has demonstrated its effectiveness in the early detection of lung cancer. Therefore, it is plausible to believe that improved image quality may contribute to a more accurate diagnosis of lung cancer in the potentially at-risk population screened over time. However, time-series studies are not the best suited to investigate the potential impact of detection bias [7, 8, 24].

The literature [15, 25] is inconsistent regarding the magnitude of radon exposure’s impact on lung cancer development, primarily due to variations in exposure propensity within study areas. Radiation carcinogenesis is a complex process [15, 25] influenced by genetic factors and environmental agents, such as housing types and inhabitant behaviours. Methodological differences and geographical variations can also affect study results. One such factor influencing our results could be heterogeneity among the municipalities studied. Previous research [16] has identified high heterogeneity in similar studies, primarily attributed to sample size, individual variations among regions and other risk factors like smoking and residential radon levels. These uncertainties may have led to an underestimation of reported results [25].

However, several factors should be considered when interpreting our findings. Due to the lack of individual data on radon exposure, lung cancer characteristics and risk factors, an ecological study was the only feasible epidemiological design for this region. This study utilised data on disease incidence, hospitalisation and mortality rates from specific geographic locations available in accessible information systems, comparing coefficients with radon exposure probabilities. Other studies investigating radon exposure and neoplasms have employed similar methodologies [26]. However, the use of aggregated data makes ecological studies susceptible to ecological fallacies, limiting their ability to infer causality [19]. Instead, they are valuable for hypothesis formulation. While the study design was not robust enough for etiological investigations, it effectively demonstrated the need for further research into radon exposure in Rio Grande do Sul.

Regarding the data source for coefficient calculation, the SIH/SUS, designed with an accounting focus [27], minimises reporting errors. While its coverage is limited to hospitals within the SUS, which represents over 70% of the Brazilian population [28], the risk of classification errors is minimal. The hospital environment enhances diagnosis accuracy, and periodic SUS audits further reduce error rates. The SIM has been collecting data since 1979. Although regional variations in data quality exist, the literature suggests continuous improvement over time [29].

Two aspects are noteworthy regarding the exposure data sources. First, direct radon measurement in municipalities is impractical, necessitating the use of an indirect measure: exposure probability. Areas with high granite or uranium-rich soil concentrations often have elevated radon levels [30]. Thus, the eU parameter was used to identify municipalities with higher radon exposure probabilities. Second, the geophysical survey was conducted in 2010. Given uranium’s extremely long half-life, its concentration in a specific area is unlikely to change significantly over time. Uranium was produced during the formation of the Earth, and takes 4.5 billion years to fully decay [17]. Future surveys may provide more detailed data due to technological advancements, but the overall uranium concentration in the assessed municipalities is expected to remain relatively constant.

While the Rio Grande do Sul Shield Aerogeophysical Project [21] is a significant and relevant study, its mapping system included only 85 of 497 municipalities in Rio Grande do Sul. Additionally, the study’s criterion of including only municipalities with 100% aerogammaspectrometry coverage significantly reduced the number of analysed municipalities, ultimately limiting it to 37. Despite the small sample size, this study’s methodological rigor in classifying municipalities by radon exposure probability has positive aspects. Given the distribution of uranium concentration on Earth [18, 22], the stratification criterion adopted, based on maximum and median eU values, provided a high level of specificity. These criteria were appropriate for selecting and classifying municipalities for comparison.

The study’s limitations include the inability to include individual variables and eliminate confounding factors. Smoking, as the primary risk factor for lung cancer, could not be controlled for in this study. However, other studies have shown that smokers have a higher incidence of lung cancer compared to non-smokers [16]. The combined effects of radon and tobacco smoke on lung cancer risk are well-documented. Tobacco smoke and radon act synergistically in the early stages of the carcinogenic process that causes lung cancer, increasing the risk of lung cancer death by 20–25 times in smokers exposed to radon levels exceeding 200 Bq/m^3^ [30].

While certain limitations were identified in this study, it is important to note its robust methodology, long historical series, standardised coefficients and rigorous selection criteria in the selection and determination of municipalities considered most susceptible to exposure, which ensured reliable results.

This study represents a pioneering investigation into the role of radon exposure in lung cancer pathogenesis in Rio Grande do Sul, Brazil. However, research on this topic is limited in many countries due to data scarcity. A recent systematic review found that no Central or South American countries have a national residential radon exposure map or unified legislative approach to address radon in homes [31]. In Brazil, the lack of residential radon data and a unified public registry of individuals with lung cancer present significant challenges for such studies. The Geological Survey of Brazil’s commitment to identifying potential radon risk areas is commendable and brings new perspectives to this topic in Brazil.

The study’s results reinforce the need to prioritise municipalities with higher radon exposure probabilities in investigations, mitigating efforts and lung cancer epidemiological surveys. This study contributes to the literature by highlighting the methodological difficulties in investigating radon exposure in relation to lung cancer pathogenesis, particularly due to the lack of direct radon measurement data in indoor environments such as homes.

## Conclusion

In conclusion, the results of this study reinforce that municipalities with a higher radon exposure probability should be prioritised for investigations and actions aimed at mitigating radon exposure and conducting epidemiological surveillance of lung cancer. This study contributes by revealing the methodological difficulties faced in investigating the relationship between radon exposure and the pathogenesis of lung cancer, particularly due to the lack of direct data on radon measurements in indoor environments, especially in residences.

## Conflicts of interest

The authors declared that they have no conflicts of interest.

## Funding

Not applicable.

## Associated academic work

This article is based on the master’s thesis ‘Radon gas and lung cancer in the State of Rio Grande do Sul: ecological study,’ completed by Jacson Cristiano Jung in the Postgraduate Program in Collective Health at the University of Vale do Rio dos Sinos (UNISINOS) in São Leopoldo, Rio Grande do Sul, Brazil. This thesis was approved on 27 March 2024.

## Ethical considerations

As this was an ecological study that analysed aggregated data without identifying individuals, approval by a research ethics committee was not required.

## Author contributions

Jung JC, Costa JSD, Oliveira CHE, Souza Filho OA and Paniz VMV contributed to the study conception and design. Jung JC contributed to data collection, processing and preliminary analyses. Jung JC, Costa JSD and Paniz VMV performed the final analyses, interpreted the data and wrote the manuscript. All authors reviewed and approved the final version of the manuscript and are responsible for all aspects, including ensuring its accuracy and integrity.

## Figures and Tables

**Table 1.** Adjusted lung cancer rates in 37 municipalities of Rio Grande do Sul (Brazil), grouped by estimated probability of radon exposure.

Adjusted lung cancer rates	Average (In the period)	95%CI ^a^	*p*-value ^b^
Incidence rate (2013–2021)			0.720
Municipalities with low exposure probability^c^	11.5 **^d^**	10.1; 12.9	
Municipalities with increased probability to exposure^c^	12.1 **^d^**	9.9; 14.3	
Hospitalization rate (2000–2021)			0.821
Municipalities with low exposure probability^c^	19.5 **^e^**	16.1; 22.9	
Municipalities with increased probability to exposure^c^	20.6 **^e^**	14.0; 27.2	
Total Mortality rate (2000–2021)			0.203
Municipalities with low exposure probability^c^	28.1 **^f^**	26.1; 30.1	
Municipalities with increased probability to exposure^c^	24.4 **^f^**	18.4; 30.4	

Legend:

a 95% Confidence interval

b *p*-value from statistical comparison between exposure groups (e.g., Mann–Whitney *U* test)

c Municipalities were grouped into two categories based on airborne gamma spectrometry data:

– *Low exposure probability*: areas with lower equivalent uranium concentrations (eU);

– *Increased exposure probability*: areas with higher eU values, indicating greater radon susceptibility.

d Incidence rate per 100,000 inhabitants

e Hospitalisation rate per 100,000 inhabitants

f Mortality rate per 100,000 inhabitants

**Table 2.** Trend in adjusted lung cancer rates in 37 municipalities of Rio Grande do Sul by radon exposure.

Adjusted lung cancer rates	Coef.^a^	95%CI^b^	*p*-value^c^	Trend
Incidence rate (2013–2021)				
Municipalities with low exposure probability^d^	0.27	−0.13; 0.68	0.153	Stationary
Municipalities with increased probability to exposure^d^	1.01	0.22; 1.81	0.020	Growing
Hospitalisation rate (2000–2021)				
Municipalities with low exposure probability^d^	0.46	0.08;0.85	0.021	Growing
Municipalities with increased probability to exposure^d^	1.15	0.70; 1.60	<0.001	Growing
Total mortality rate (2000–2021)				
Municipalities with low exposure probability^d^	−0.16	−0.33; 0.01	0.058	Stationary
Municipalities with increased probability to exposure^d^	0.02	−0.33; 0.37	0.899	Stationary

Caption:

a Prais–Winsten regression coefficient indicating the average annual change in the rate

b 95% Confidence Interval (CI) for the regression coefficient

c Statistical significance of the trend, based on the Prais–Winsten regression

d Municipalities grouped based on estimated radon exposure probability derived from airborne gamma spectrometry (eU concentrations):

– *Low exposure probability*: lower eU levels

– *Increased exposure probability*: higher eU levels, indicating greater radon susceptibility
